# Macular dystrophies associated with Stargardt-like phenotypes

**DOI:** 10.5935/0004-2749.2021-0415

**Published:** 2023-03-08

**Authors:** Rebeca A. S. Amaral, Olivia A. Zin, Mariana V. Salles, Fabiana L. Motta, Juliana Maria Ferraz Sallum

**Affiliations:** 1 Department of Ophthalmology and Visual Sciences, Universidade Federal de São Paulo, São Paulo, Brazil; 2 Instituto de Genética Ocular, São Paulo, Brazil

**Keywords:** Stargardt disease, Genetic association studies, Phenotype, Inheritance patterns, High-throughput nucleotide sequencing, Macular degeneration, Retinal dystrophies, Genetic diseases, Doença de Stargardt, Estudos de associação ge-nética, Fenótipo, Padrões de herança, Sequenciamento de nucleotídeos em larga escala, Degeneração macular, Distrofias retinianas, Doenças genéticas

## Abstract

**Purpose:**

Stargardt-like phenotype has been described as associated with pathogenic variants besides the *ABCA4* gene. This study aimed to describe four cases with retinal appearance of Stargardt disease phenotypes and unexpected molecular findings.

**Methods:**

This report reviewed medical records of four patients with macular dystrophy and clinical features of Stargardt disease. Ophthalmic examination, fundus imaging, and next-generation sequencing were performed to evaluate pathogenic variants related to the phenotypes.

**Results:**

Patients presented macular atrophy and pigmentary changes suggesting Stargardt disease. The phenotypes of the two patients were associated with autosomal dominant inheritance pattern genes (*RIMS1* and *CRX*) and in the other two patients were associated with recessive dominant inheritance pattern genes (*CRB1* and *RDH12*) with variants predicted to be pathogenic.

**Conclusion:**

Macular dystrophies may have phenotypic similarities to Stargardt-like phenotype associated with other genes besides the classic ones.

## Abstract (pt)

**Objetivo:**

Fenótipos Stargardt-like já foram asso-ciados a variantes patogênicas no gene *ABCA4*. O propósito desse estudo é descrever quatro pacientes com achados retinianos semelhantes a doença de Stargardt com resultados moleculares diferentes do esperado.

**Métodos:**

Esse relato fez a revisão de prontuários médicos de quatro pacientes com distrofia macular e achados clínicos sugestivos de doença de Stargardt. Foram realizados avaliação oftalmológica, exames de imagens e testes usando *next generation sequencing* para avaliar variantes patogênicas associadas aos fenótipos dos pacientes.

**Resultados:**

Os pacientes apresentavam atrofia macular e alterações pigmentares sugerindo achados clínicos de doença de Stargardt. Dois pacientes foram associados a genes com herança autossômica dominante (*RIMS1* e *CRX*) e dois pacientes foram associados a genes com herança autossômica recessiva (*CRB1* e *RDH12*) com variantes preditoras de serem patogênicas.

**Conclusão:**

Distrofias maculares podem ter similaridades fenotípicas com fenótipo de Stargardt-like associados a outros genes além dos classicamente já descritos.

## INTRODUCTION

Stargardt disease (STGD1, OMIM #248200) is one of the most frequent causes of inherited macular dystrophy^(1)^. It manifests mainly during childhood and teenager years^(2^-^3)^; however, onset in early adulthood was also reported^(3^-^4)^. Patients present with central visual loss, loss of color vision, photophobia, and paracentral scotoma^(1^-^5)^. The disease leads to the loss of the external segments of the photoreceptors and retina pigmentary epithelium (RPE) cells^(5^-^6)^, with lipofuscin deposits causing flecks at the level of the RPE^(5^-^6)^. Fundoscopy reveals bilateral yellow-white flecks deposits on the macula, which evolve to chorioretinal macular atrophy. In advanced stages, the disease may spread throughout the posterior pole^(5^-^6)^.

However, STGD1 has much broader phenotypical spectrum, which includes macular atrophy without flecks, bull’s-eye maculopathy-like phenotype, fundus flavimaculatus (flecks without atrophy), foveal-sparing phenotype, cone-rod dystrophy (CORD), and retinitis pigmentosa (RP)-like phenotype^(7)^. Disease progression also varied, but patients with early childhood onset typically have a more severe phenotype and more rapid disease progression^(3)^. By contrast, patients with a late-onset disease (>45 years of age) usually have a milder phenotype and slower progression^(4)^.

STGD1 autosomal recessive form is usually related to biallelic variations in the *ABCA4* gene (OMIM *601691), but *PRPH2* (OMIM *179605), *PROM1* (OMIM *604365), and *ELOVL4* (OMIM *605512) may also cause similar phenotypes. The clinical appearance of autosomal dominant (AD) Stargardt-like macular dystrophy is similar to the *ABCA4* autosomal recessive (AR) phenotype, making it difficult to differentiate by fundus examination alone^(8)^.

Retinal disorders with clinical phenotypes resembling STGD1 but with a dominant pattern of inheritance are referred to as “Stargardt-like.” This study aimed to describe four cases that have Stargardt-like phenotype, whose molecular diagnosis reveal mutations in genes other than those classically associated with STGD1.

## METHODS

This retrospective study assessed medical records of four Brazilian patients examined at *Instituto de Genética Ocular in São Paulo*, Brazil. All patients had macular dystrophy compatible with the clinical diagnosis of STGD1. They had STDG1 as one of the clinical diagnosis hypothesis, but the identified gene was other than *ABCA4, PRPH2, PROM1,* or *ELOVL4.*

The molecular genetic data obtained from commercial tests was performed by a next-generation sequencing Illumina system (Illumina, San Diego, CA, USA) panel with 224 genes related to the inherited retinal dystrophies.

This study was approved by the Ethics Committee in Research of *Universidade Federal de São Paulo (Protocol #6159)*. All patients provided written informed consent for the use of personal medical data for scientific purposes and publication. This study was also performed in accordance with the ethical standards of the 1964 Declaration of Helsinki and its subsequent amendments.

For appropriate classification of pathogenicity, popu-lation databases [Genome Aggregation Database (gnomAD), Exome Aggregation Consortium (ExAC), and 1000 Genomes Project)] and human variation and phenotype databases [(ClinVar), Universal Protein Resource (UniProt), and Human Gene Mutation Database] were consulted. Evaluation variants were made according to American College of Medical Genetics (ACMG) standardization.

## RESULTS

All four patients had clinical features of STGD, such as progressive central visual loss, bilateral flecks or fundus flavimaculatus, pigmentary changes, and macular atrophy. Their visual acuity ranged from 20/30 to 20/200. All patients presented onset of symptoms at age 40s or later, except for patient 3 who presented at teenager years. Consanguinity was not reported in any case.

Table 1 shows the results of molecular tests and clinical findings. Two patients presented AD pattern of inheritance (patients 1 and 2), and two patients presented AR pattern of inheritance (patients 3 and 4). Segregation analyses were performed only for patients 1 and 3, and it confirmed the inheritance presentation (Table 2). *ABCA4* was fully sequenced and presented no pathogenic variant in any of the patients.

**Table 1:** Clinical data and genetic findings

Patient	Sex	Age of onset (years)	Visual acuity (RE; LE)	Symptoms at the time of diagnosis	Fundus examination		Allele 1		Allele 2		
						Gene	Nucleotide change	Protein change	Nucleotide change	Protein change	Inheritance pattern
1	F	40	20/40; 20/40	Delayed dark adaptation and nyctalopia	Macular flecks and pigmentary changes	*RIMS1*	c.2093G>A	p.Arg698Gln	-	-	AD
2	M	48	20/200; 20/30	Loss in central vision in the RE	Macular atrophy in the RE and bull’s eye in the LE	*CRX*	c.121C>T	p.Arg41Trp	-	-	AD
3	M	12	20/60; 20/60	Delayed dark adaptation and loss in central vision	Yellow deposits in the macular area and pigmentary changes	*CRB1*	c.2843G>A	p.Cys948Tyr	c.408_506del	p.Ile167_Gly169del	AR
4	F	40	20/60; 20/60	Photophobia, loss in central vision	Macular and paramacular atrophy	*RDH12*	c.295C>A	p.Leu99Ile	c.700C>T	p.Arg234Cys	AR

Female, F; male= M; RE= right eye; LE= left eye; AD= autosomal dominant; AR= autosomal recessive.

**Table 2:** Segregation analysis from #1 and #3

Patient		Fundus examination	Gene	Allele 1		Allele 2	
				Nucleotide change	Protein change	Nucleotide change	Protein change
1	Daughter	Macular atrophy and pigmentary changes	*RIMS1*	c.2093G>A	p.Arg698Gln	-	-
3	Mother	Narrow vessels and macula edema	*CRB1*	c.613_619del	p.Ile205Asp fs^*^13	c.408_506del	p.Ile167_Gly169del

**Figure 1:**
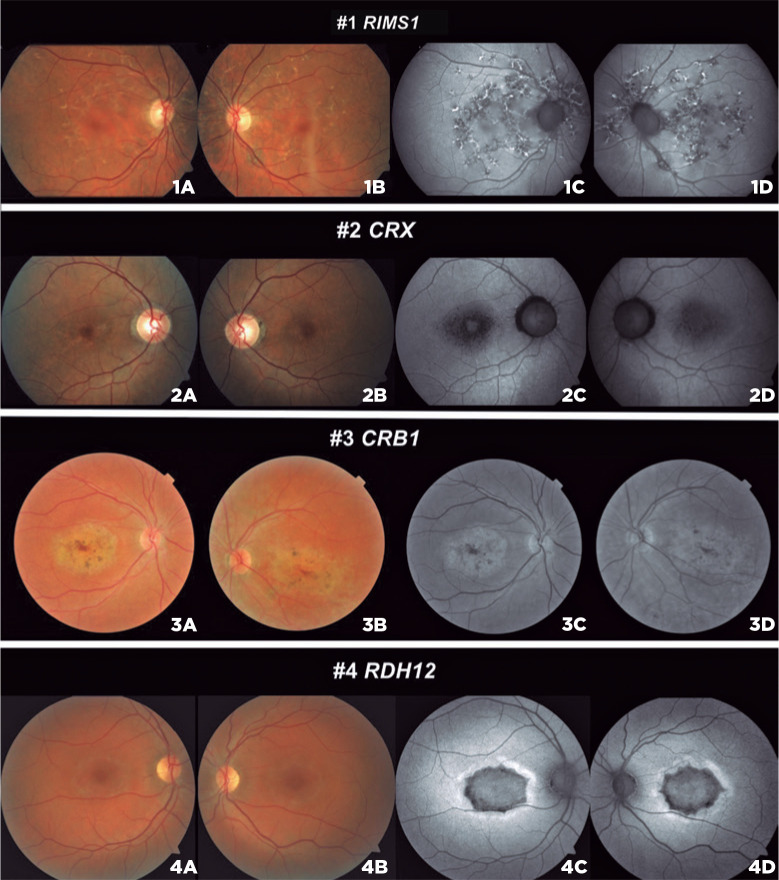
Retinal image findings. (1A, 1B) *RIMS1* patient color fundus of the RE and LE and (1C, 1D) FAF photographs showing flecks in the posterior pole. (2A, 2B) *CRX* patient color fundus and (2C, 2D) FAF with bull’s eye appearance in the macula in both eyes. (3A, 3B) *CRB1* patient with yellow deposits and (3C, 3D) red free-fundus photographs with symmetrical hyperfluorescence atrophy in the macula. (4A, 4B) *RDH12* patient macula atrophic changes and (4C, 4D) macula hypoautofluorescence surrounded with hyperautoflourescence halo. LE, left eye; RE, right eye.

**Figure 2:**
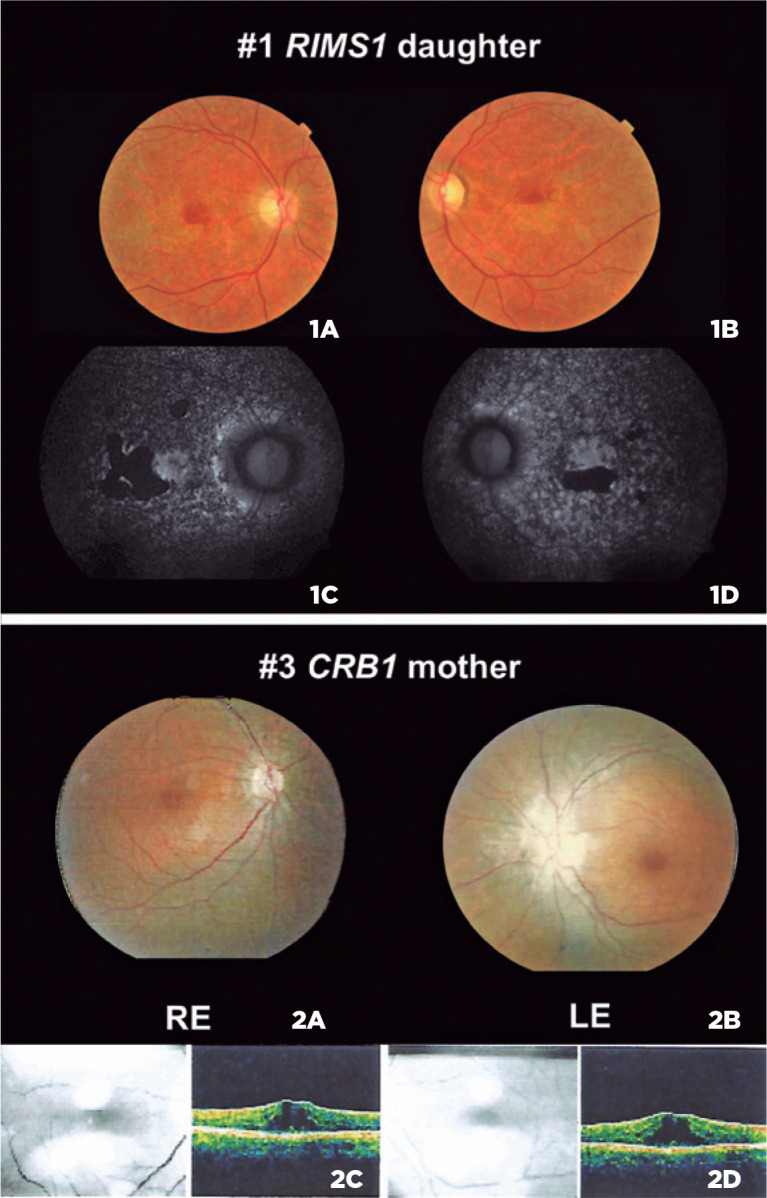
(1A, 1B) Color fundus image from *RIMS1* patient’s daughter and FAF (1C, 1D) from the RE and LE. (2A, 2B) Color fundus image from *CRB1* patient’s mother and macula OCT (2C, 2D) from the RE and LE. LE, left eye; RE, right eye.

Patient 1 presented with flecks around the macula and posterior pole, extending beyond the arcades and nasally to the optic disc, as well as pigmentary changes (Figure 1, 1A-1D). All findings were symmetrical in both eyes. Fundus autofluorescence (FAF) examination revealed hyperand hypoautofluorescent point areas. The molecular test of patient 1 presented heterozygous variant p.Arg698Gln in the *RIMS1* (OMIM *606629) gene. This variant is classified as of uncertain significance (VUS) and has never been reported previously. The population frequency of this variant is >1% (gnomAD; f=0.0000121). For appropriate classification of pathogenicity, several mutation prediction software programs considered this variant probably pathogenic (DANN, DEOGEN2, EIGEN, FATHMM-MKL, M-CAP, MutationAssessor, MutationTaster, PrimateAI, and SIFT). *RIMS1* gene is associated with an AD trait. The proband’s parents were unavailable for segregation analysis, but the patient’s daughter was had a milder phenotype and presented the same variant in this gene (Figure 2).

Patient 2 had macular atrophy, and FAF exam revealed an area of foveal hypoautofluorescence surrounded by a retina with a homogeneous appearance, typically seen in patients with STGD (Figure 1, 2A-2D). Genetic sequencing presented the heterozygous variant p.Arg41Trp in *CRX* (OMIM *602225) gene classified as likely pathogenic according to ACMG criteria. This variant has been associated with inherited retinal dystrophies with AD pattern^(9)^. The patient’s brother presented the same symptoms but was unavailable for segregation analysis.

Patient 3 had yellow vitelliform deposits in the macular area associated with pigmentary changes, suggesting advanced disease (Figure 1 3A-3D). Although the first symptoms were present early in life, no nystagmus was reported in this case. He was a compound heterozygous for the *CRB1* (OMIM *604210) gene presenting a missense variant p.Cys948Tyr and a deletion p.Asp165_Ile167del (likely pathogenic). The variant p.Cys948Tyr has been described as definitely pathogenic in the literature^(10)^. Homozygous null alleles or homozygous p.Cys948Tyr alleles are found in Leber congenital amaurosis (LCA), early-onset retinal dystrophy (EORD), and RP^(11)^. The protein change p.Ile167_Gly169del has been previously reported to be associated with retinopathy and classified as a likely pathogenic variant^(12^-^13)^. The patient’s mother presented the same deletion variant in *CRB1,* fundus presentation with pigmentary changes, narrow vessels, macula edema, and late-onset symptoms (Figure 2).

Patient 4 had extensive atrophic changes in the macula and paramacular areas associated with pigmentary deposits. The FAF exam revealed macular hypoautofluo-rescence atrophy in both eyes (Figure 1 4A-4D). She had two heterozygous variants in the *RDH12* (OMIM *608830) gene: p.Leu99Ile has been described as pathogenic in LCA in the compound heterozygous state^(14)^ and p.Arg234Cys classified as VUS that has never been reported previously. The latter is a rare variant with <1% in population frequency (gnomAD; f=0.00000402) and is considered damaging or pathogenic in an *in silico* analysis that predicts the effects of protein missense mutations (FATHMM, SIFT, PROVEAN, MVP, MetaSVM, and MetaLR).

## DISCUSSION

This study was conducted to describe four patients with phenotype findings that do resemble Stargardt-like features. Clinical diagnosis is difficult because these disorders are linked to different genes that lead to the same clinical phenotypes.

The four patients presented herein have phenotypes similar to STGD1. Each patient had particularities that differentiate from this phenotype, which can complicate the diagnosis and underline the need for accurate genetic testing. Table 3 summarizes these clinical findings.

**Table 3:** Clinical similarities and differences from STGD1

Patient	Findings that suggest STGD1	Findings that differentiate from STGD1
1	Flecks around the macula and posterior pole	AD inherited pattern
2	Foveal hypoautofluorescence surrounded by a homogeneous retina	Asymmetric VA
3	Macular atrophy and pigmentary changes	Cystoid macular edema in te retina of the mother
4	Macular atrophy and preserved vessels	Different hypoautofluorescence macular aspect in FAF

Genetically, patient 1 presented the heterozygous variant p.Arg698Gln in the *RIMS1* gene. This gene expression is limited to the brain and retina, localized in the presynaptic active zone^(15)^, and interacts with Rab3A, a protein known to regulate synaptic vesicle exocytosis, suggesting that it may be essential in regulating neurotransmitter release^(16)^.

This patient presented flecks around the macula and posterior pole and hyper and hypoautofluorescence areas in the FAF exam that suggest STGD1. The family history leads to an AD inheritance because the patient’s daughter presented bilateral mild decrease in visual acuity with yellow deposits on the macula and hypoautofluorescence area corresponding to macular atrophy. It was a different fundus aspect compared with her mother and revealed AD pattern of inheritance.

The genetic results reinforce this AD hypothesis because the *RIMS1* gene is associated with AD CORD7 (#OMIM 603649). The segregation analysis revealed that the patient’s daughter presented the same variant and and multiple lines of computational evidence support a deleterious effect on the gene supporting pathogenicity for p.Arg698Gln variant. This may explain the phenotype exhibited by this patient.

To date, the CORD7 phenotype has only been described in eight members of a four-generation, non-consanguineous British family, which had a missense variant in the *RIMS1* gene (p.Arg844His) as the disease-causing mutation^(16^-^18)^. Most of these individuals experienced progressive worsening of central vision, nyctalopia, and peripheral visual field loss between the third and fourth decades of life^(18)^. Visual acuity ranged between 20/20 and 20/400, whereas fundus changes varied from mild RPE changes to extensive atrophy and pigmentation. In the majority of individuals, FAF examination showed decreased central autofluorescence with a surrounding ring of increased autofluorescence^(18)^.

In patient 2, the heterozygous variant p.Arg41Trp was the causative genetic error in the *CRX* gene. Previous studies have implicated *CRX* in dominant forms of CORD^(19^,^20)^ RP^(19)^, and (LCA)^(20)^. The *CRX* gene possibly plays a role in both early and late photoreceptor development, since its expression in the mouse retina begins at the time of cone cell genesis, and its peak expression is near the time of maximal rod cell proliferation and genesis. Nonetheless, it is also highly expressed in adult retina^(19)^. It controls outer-segment photoreceptor biogenesis and disk renewal by binding and transactivating the genes for several photoreceptor cell-specific proteins found in major outer-segment photoreceptor proteins (such as interphotoreceptor retinoid-binding protein, β-phosphodiesterase, arrestin, and rhodopsin)^(21)^. Consequently, *CRX* mutations may reduce the synthesis of important outer-segment photoreceptor proteins, which is associated with photoreceptor degeneration^(9)^.

Rivolta et al. listed 18 *CRX* mutations, including p.Arg41Trp, all of which caused disease with an AD pattern and complete penetrance^(22)^. The diagnosis can vary even among patients with the same primary mutation. This was shown by Hull et al. in a series of 19 patients from 11 families with *CRX* mutations ^(23)^. Four families demonstrated a wide intrafamilial phenotypic heterogeneity, with different clinical diagnosis in individuals with the same mutation, such as LCA, CORD, cone dystrophy, and a novel macular phenotype. The heterogeneity of clinical phenotype among those sharing the same *CRX* mutant allele could be caused by the influence of polymorphisms in the *CRX* promoter region, polymorphisms in co-expressed transcription factors, or effect of environmental factors^(23)^.

Nishiguchi et al. established a genotype-phenotype correlation according to the location of the *CRX* mutation, mutations in the homeobox domain, positions 39 to 99, which were more likely to cause macular dystrophy^(24)^. On the contrary, mutations downstream to the homeobox domain were associated with a more severe phenotype, described as macular or pan-retinal degeneration with bone spicules^(24)^. This association is corroborated here becuase mutation p.Arg41Trp led to macular dystrophy with no pan-retinal degenerations and bone spicules.

Patient 3 had a compound heterozygous variant at the *CRB1* gene, a missense variant p.Cys948Tyr, and a deletion p.Ile167_Gly169del. The missense variant p.Cys948Tyr has been described as definitely pathogenic in the literature and associated with LCA, EORD, and RP in an AR pattern^(25)^. Mutations in the *CRB1* gene lead to retinal abnormalities such as thickening, coarse lamination patterns, and loss of photoreceptor signaling^(25)^. Bujakowska et al. suggested a possible association between the severity of the variant and the phenotype^(11)^, and it was also reported by our group (Motta et al.) in another publication^(12)^. Patients with milder inherited retinal dystrophies have missense variants or in-frame deletions, and patients with more severe phenotypes, for example, macular atrophy, tend to have protein truncation (nonsense or frameshift deletions) and/or p.Cys948Tyr variants^(11^,^12)^. The second allele variant was a deletion, and this might explain the onset symptoms at the age of 12 years and the milder loss of visual acuity. In the patient’s mother, symptom onset was at around the age of 37 years, presenting with pigmentary changes in the posterior pole, narrow vessels associated with macular edema, and typical findings in RP. She also had a compound heterozygous variant in *CRB1* presenting the same deletion found in her son, and her second allele was a frameshift variant p.Ile205Asp classified as definitely pathogenic, which was different from the second allele variant on her son.

The modifying effects of non-genetic factors (e.g., environmental) were suggested as a reason for phenotype variation in *CRB1* dystrophy^(11)^. Patient 3 presented a *ABCA4* gene variant p.Arg2107His classified as VUS, in which multiple lines of computational evidence support a deleterious effect on the gene or gene product (conservation, evolutionary, splicing effect, etc.). This variant has a population frequency of >1% (gnomAD; f=0.00148) and segregated with the patient’s mother genotype. Mutations that affect *CRB1* and *ABCA4* segregating with two different phenotypes in the same family was previously described^(26)^, and a modulating effect was suspected.

Patient 4 presented the *RDH12* variant p.Leu99Ile, which was previously reported as pathogenic in compound heterozygous state in LCA^(14)^. The second allele variant was p.Arg234Cys, which was considered damaging in the *in silico* analysis, although it has a low conservation score. This might be associated with the possible pathogenic effect in the protein function that can lead to a late-onset clinical presentation.

In patient 4, fundus examination revealed retinal atrophy in the macular region surrounded by a hyperautofluorescent halo that may suggest STGD. No pathogenic variant was found in *ABCA4*. AR *RDH12* retinopathy usually present in infancy with early-onset visual loss^(27)^. AD *RDH12* retinopathy was found in a six-generation family with 19 affected members, presenting with a late-onset RP phenotype, intraretinal bone spicule pigmentation, and arteriolar attenuation^(28)^. *RDH12* encodes retinol dehydrogenase 12, an enzyme expressed in photoreceptors that reduce all-*trans*-retinal to all-*trans*-retinol^(29)^. The clearance of all-*trans*-retinal consists of two steps: translocation of all-*trans*-retinal across the photoreceptor disc membranes by ATP-binding cassette transporter 4 (ABCA4) and reduction of all-*trans*-retinal to all-*trans*-retinol by retinol dehydrogenase 8 (RDH8) expressed in the outer segments of photoreceptors and RDH12 located in photoreceptor inner segments^(29)^. Impaired removal of all-*trans*-retinal from photoreceptors was suggested as an important mechanism involved in retinal degeneration^(30)^.

While currently no treatments are commercially available for STGD1, several categories of therapeutics are under investigation to potentially find this outcome. The pharmacological modulation of the visual cycle serves as a novel approach to the potential treatment of degenerative retinal diseases. Finding the involved genes in the phenotypes leads to new possibilities of discovering treatments by increasing or decreasing the function on the metabolic pathways of those genes. As the pathophysiology of STGD1 is complex, a multi-targeted approach could help in the identification of alternative pathways or modification factors involving the disease mechanism.

In this report of four patients with macular dystrophy and history suggesting Stargardt-like disease, two patient’s phenotypes were related to AD genes (*RIMS1* and *CRX*) and those of the other two patients were related to AR genes (*CRB1* and *RDH12*).

STGD1 is the most common inherited macular dystrophy but has a wide clinical spectrum, and several inherited macular dystrophies have phenotypic similarities that can make clinical diagnosis challenging. As the disease progress, clinical appearance may change over time, and its end-stage appearance of diffuse atrophy and peripheral involvement are almost indistinguishable from each other. Functional tests are still important for the characterization of the phenotype and help in the diagnostic definition, especially in cone dystrophies, which are often the main differential diagnosis for STGD1.

Molecular genetic studies and detailed clinical descriptions have demonstrated that a central atrophic lesion with surrounding subretinal yellow flecks can arise secondary to mutations in different genes. With the improvement of potential treatments for inherited retinal dystrophies, correct molecular diagnosis is essential.
